# 1178. Sustained Vaccine Effectiveness Against Influenza-Associated Hospitalization in Children: Evidence from the New Vaccine Surveillance Network, 2015-2016 Through 2019-2020

**DOI:** 10.1093/ofid/ofab466.1371

**Published:** 2021-12-04

**Authors:** Leila C Sahni, Eric A Naioti, Samantha M Olson, Angela P Campbell, Marian G Michaels, John V Williams, Mary Allen Staat, Elizabeth P Schlaudecker, Natasha B Halasa, Natasha B Halasa, Laura S Stewart, Janet A Englund, Eileen J Klein, Peter G Szilagyi, Geoffrey A Weinberg, Christopher J Harrison, Rangaraj Selvarangan, Parvin H Azimi, Monica Nayakwadi Singer, Pedro Piedra, Flor M Munoz, Manish Patel, Julie A Boom

**Affiliations:** 1 Texas Children’s Hospital, Houston, Texas; 2 Centers for Disease Control and Prevention (CDC), Binghamton, New York; 3 CDC, Atlanta, Georgia; 4 Centers for Disease Control and Prevention, Atlanta, GA; 5 Children’s Hospital of Pittsburgh of UPMC, Pittsburgh, PA; 6 University of Pittsburgh, Pittsburgh, Pennsylvania; 7 Cincinnati Children’s Hospital Medical Center, Cincinnati, OH; 8 Cincinnati Children’s Hospital Medical Center, University of Cincinnati College of Medicine, Cincinnati, OH; 9 Vanderbilt University Medical Center, Nashville, TN; 10 Seattle Children’s Hospital/Univ. of Washington, Seattle, Washington; 11 Seattle Children’s Hospital, Seattle, Washington; 12 University of California, Los Angeles, Los Angeles, California; 13 University of Rochester, Rochester, New York; 14 Children’s Mercy Hospital, Kansas City, MO; 15 UCSF, Berkeley, CA; 16 UCSF Benioff Children’s Hospital Oakland, Lafayette, CA; 17 Baylor College of Medicine, Houston, TX

## Abstract

**Background:**

Adult studies have demonstrated intra-season declines in influenza vaccine effectiveness (VE) with increasing time since vaccination; however, data in children are limited.

**Methods:**

We conducted a prospective, test-negative study of children ages 6 months through 17 years hospitalized with acute respiratory illness at 7 pediatric medical centers each season in the New Vaccine Surveillance Network during the 2015-2016 through 2019-2020 influenza seasons. Cases were children with an influenza-positive molecular test; controls were influenza-negative children. Controls were matched to cases by illness onset date using 3:1 nearest neighbor matching. We estimated VE [100% x (1 – odds ratio)] by comparing the odds of receipt of ≥ 1 dose of influenza vaccine ≥ 14 days before the onset of illness that resulted in hospitalization among influenza-positive children to influenza-negative children. Changes in VE over time between vaccination date and illness onset date during each season were estimated using multivariable logistic regression models.

**Results:**

Of 8,430 hospitalized children (4,781 [57%] male; median age 2.4 years), 4,653 (55%) received ≥ 1 dose of influenza vaccine. On average, 48% and 85% of children were vaccinated by the end of October and December, respectively. Influenza-positive cases (n=1,000; 12%) were less likely to be vaccinated than influenza-negative controls (39% vs. 61%, p< 0.001) and overall VE against hospitalization was 53% (95% CI: 46%, 60%). Pooling data across 5 seasons, the odds of any influenza-associated hospitalization increased 0.96% (95% CI: -0.76%, 2.71%) per week with a corresponding weekly decrease in VE of 0.45% (p=0.275). Odds of hospitalization with time since vaccination increased 0.66% (95% CI: -0.76%, 2.71%) per week in children ≤ 8 years (n=3,084) and 2.16% (95% CI: -1.68%, 6.15%) per week in children 9-17 years (n=771). No significant differences were observed by virus subtype or lineage.

Figure 1. Declines in influenza VE over time from 2015-2016 through 2019-2020, overall (a) and by age group (b: ≤ 8 years; c: 9-17 years)

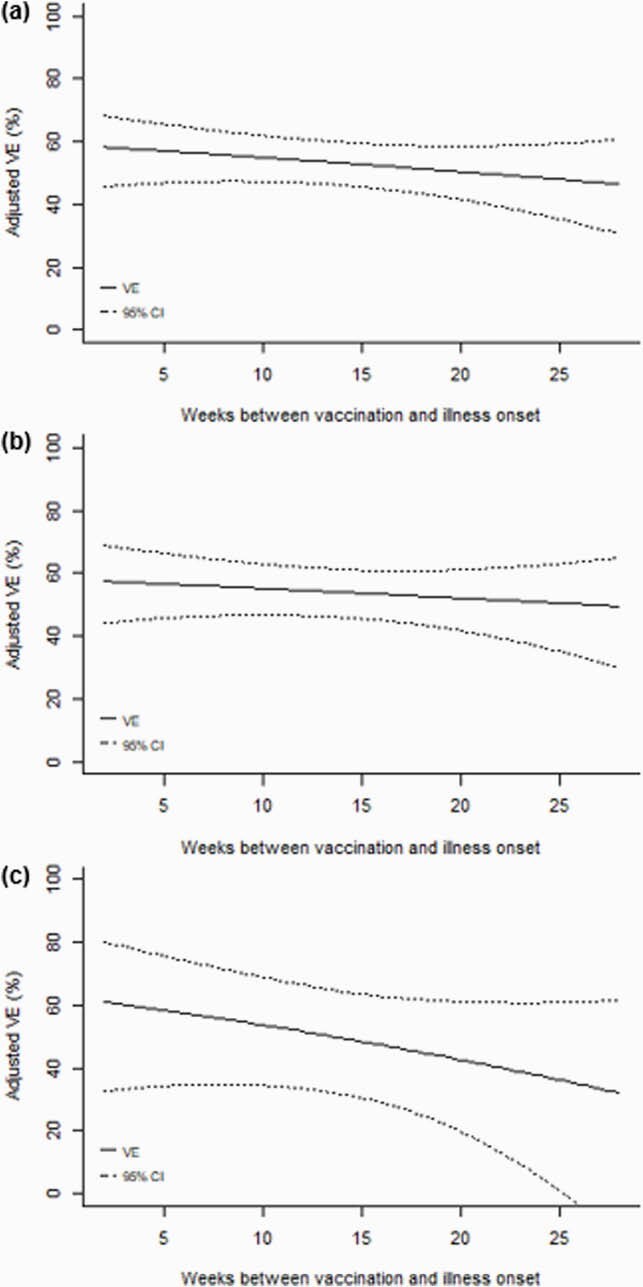

**Conclusion:**

We observed minimal intra-season declines in VE against influenza-associated hospitalization in U.S. children. Vaccination following Advisory Committee on Immunization Practices guidelines and current timing of vaccine receipt is the best strategy for prevention of influenza-associated hospitalization in children.

**Disclosures:**

**Marian G. Michaels, MD, MPH**, **Viracor** (Grant/Research Support, performs assay for research study no financial support) **John V. Williams, MD**, **GlaxoSmithKline** (Advisor or Review Panel member, Independent Data Monitoring Committee)**Quidel** (Advisor or Review Panel member, Scientific Advisory Board) **Elizabeth P. Schlaudecker, MD, MPH**, **Pfizer** (Grant/Research Support)**Sanofi Pasteur** (Advisor or Review Panel member) **Natasha B. Halasa, MD, MPH**, **Genentech** (Other Financial or Material Support, I receive an honorarium for lectures - it’s a education grant, supported by genetech)**Quidel** (Grant/Research Support, Other Financial or Material Support, Donation of supplies/kits)**Sanofi** (Grant/Research Support, Other Financial or Material Support, HAI/NAI testing) **Natasha B. Halasa, MD, MPH**, Genentech (Individual(s) Involved: Self): I receive an honorarium for lectures - it’s a education grant, supported by genetech, Other Financial or Material Support, Other Financial or Material Support; Sanofi (Individual(s) Involved: Self): Grant/Research Support, Research Grant or Support **Janet A. Englund, MD**, **AstraZeneca** (Consultant, Grant/Research Support)**GlaxoSmithKline** (Research Grant or Support)**Meissa Vaccines** (Consultant)**Pfizer** (Research Grant or Support)**Sanofi Pasteur** (Consultant)**Teva Pharmaceuticals** (Consultant) **Christopher J. Harrison, MD**, **GSK** (Grant/Research Support)**Merck** (Grant/Research Support)**Pfizer** (Grant/Research Support, Scientific Research Study Investigator, Research Grant or Support) **Flor M. Munoz, MD**, **Biocryst** (Scientific Research Study Investigator)**Gilead** (Scientific Research Study Investigator)**Meissa** (Other Financial or Material Support, DSMB)**Moderna** (Scientific Research Study Investigator, Other Financial or Material Support, DSMB)**Pfizer** (Scientific Research Study Investigator, Other Financial or Material Support, DSMB)**Virometix** (Other Financial or Material Support, DSMB)

